# Supplementary material for: Enhanced Delayed Fluorescence in Nonlocal Metasurfaces: The Role of Electronic Strong Coupling
Source: ACS Photonics. 2025 Mar 25;12(4):2193–202. doi: 10.1021/acsphotonics.5c00124 (PMC12007117; doi:10.1021/acsphotonics.5c00124)
Supplement: Supplementary file 1 — ph5c00124_si_001.pdf [file ph5c00124_si_001.pdf]

# Supporting Information: Enhanced Delayed Fluorescence in Non-Local Metasurfaces: The Role of Electronic Strong Coupling

Yu-Chen Wei,*,† Chih-Hsing Wang,‡ Konstantinos S. Daskalakis,¶ Pi-Tai Chou,‡ Shunsuke Murai,§ and Jaime Gómez Rivas*,†

†*Department of Applied Physics and Science Education, Eindhoven University of Technology, 5600MB Eindhoven, The Netherlands*

‡*Department of Chemistry, National Taiwan University, 106319 Taipei, Taiwan*

¶*Department of Materials Engineering, University of Turku, FI-20014 Turun yliopisto, Finland*

§*Department of Material Chemistry, Graduate School of Engineering, Kyoto University, Kyoto, 615-8510, Japan*

E-mail: y.c.wei@tue.nl; j.gomez.rivas@tue.nl

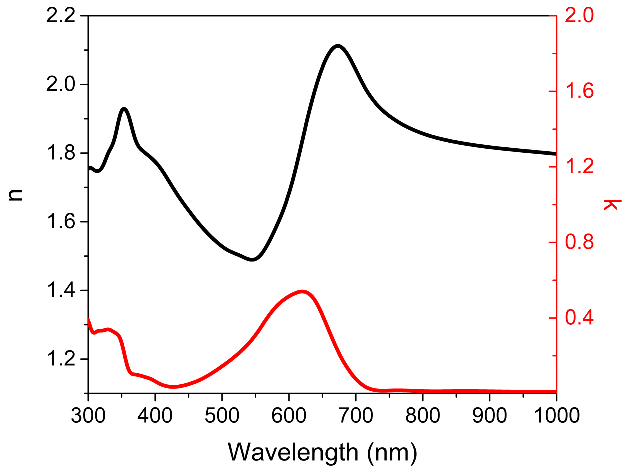


Figure S1: Complex refractive index of 50 wt% CBP/$BF_2$.

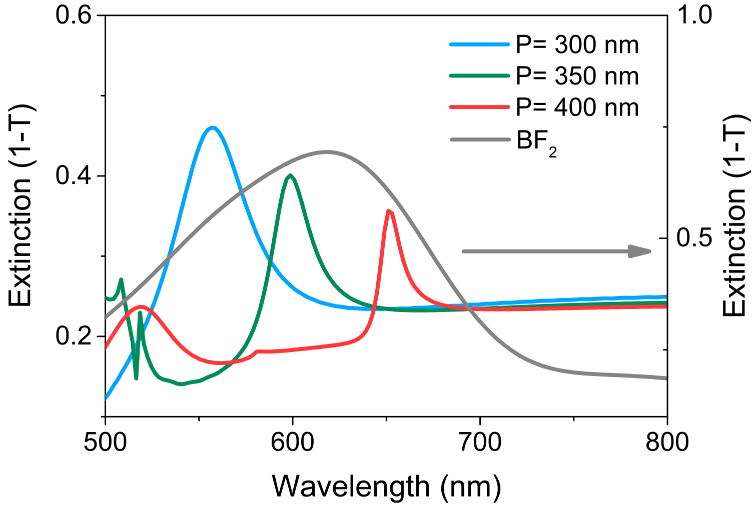


Figure S2: Simulated extinction spectra of bare arrays at normal incidence.

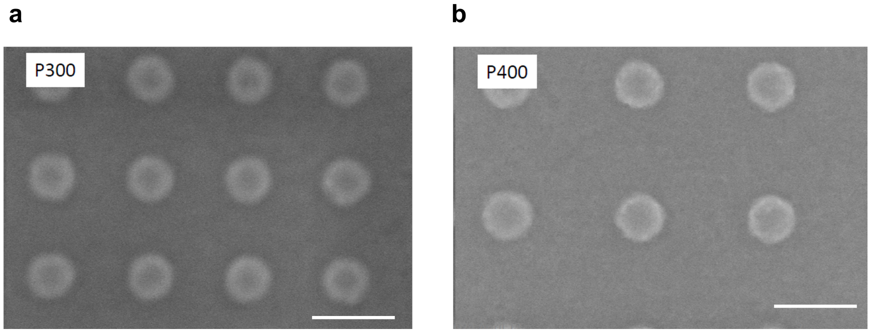


Figure S3: Scanning electron microscope images of (a) R-detuned and (b) B-detuned metasurfaces. The scale bars represent 250 nm.

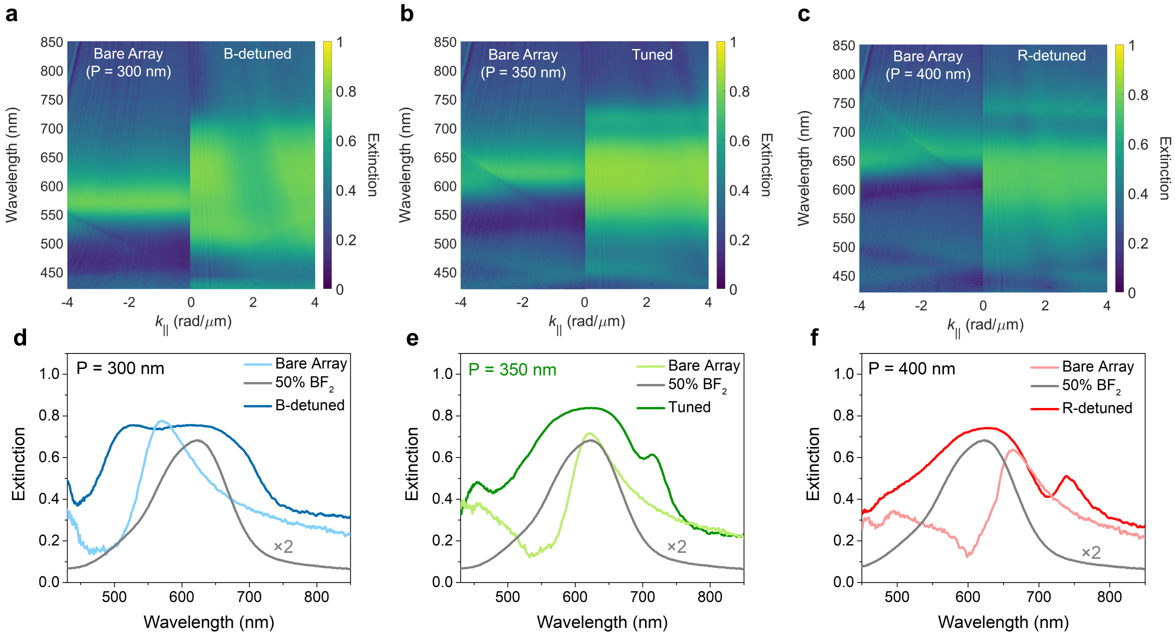


Figure S4: Angle-resolved optical extinction measurements of the tuned and detuned systems with TM illustration. Extinction maps of (a) the B-detuned system (P = 300 nm); (b) the tuned system (P = 350 nm), (c) the R-detuned system (P = 400 nm). The left parts of (a)-(c) correspond to the extinction maps of the bare arrays (CBP thin films on arrays). The right parts of (a)-(c) correspond to the extinction maps of 50 wt% CBP/$BF_2$ blended thin film on the tuned and detuned metasurfaces. Extinction spectra of (d) the B-detuned system, (e) the tuned system and (f) the R-detuned system. All spectra are taken at $k_{||}$ = 1 rad/$\mu$m. The gray lines in (d), (e) and (f) represent the emission spectra of 50 wt% CBP/$BF_2$ blended thin film (non-cavity system).

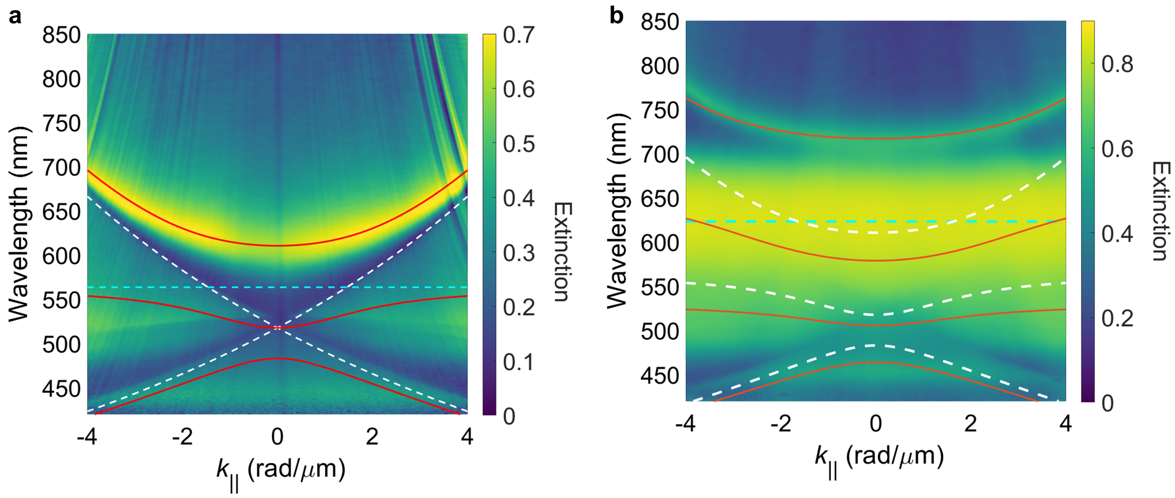


Figure S5: Fits of the coupled oscillator model to the extinction measurements. (a) Bare array (P = 350 nm), and (b) the tuned system. In (a), the white dashed lines represent the Rayleigh anomalies of the TE (±1,0) diffraction orders. The cyan dashed line indicates the localized resonance of the individual Al nanoparticles. The red solid lines are the surface lattice resonance modes, which are the eigenmodes of the Hamiltonian in Eq. 4 in the manuscript. In (b), the white dashed lines are the surface lattice resonance modes. The cyan dashed line indicates the excitonic transition from the 50 wt% CBP/$BF_2$ thin film. The red solid lines are the polaritonic modes, which are the eigenmodes of the Hamiltonian in Eq. 1 in the manuscript.

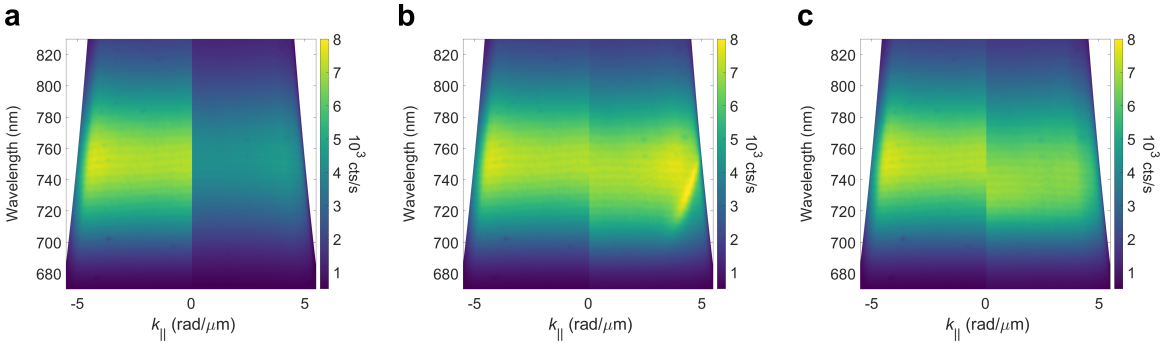


Figure S6: Angle-resolved TM-polarized PL of the tuned and detuned systems. Steady-state PL maps of the (a) B-detuned, (b) tuned and (c) R-detuned systems. The left panels of (a)-(c) correspond to the PL map of the non-cavity system.

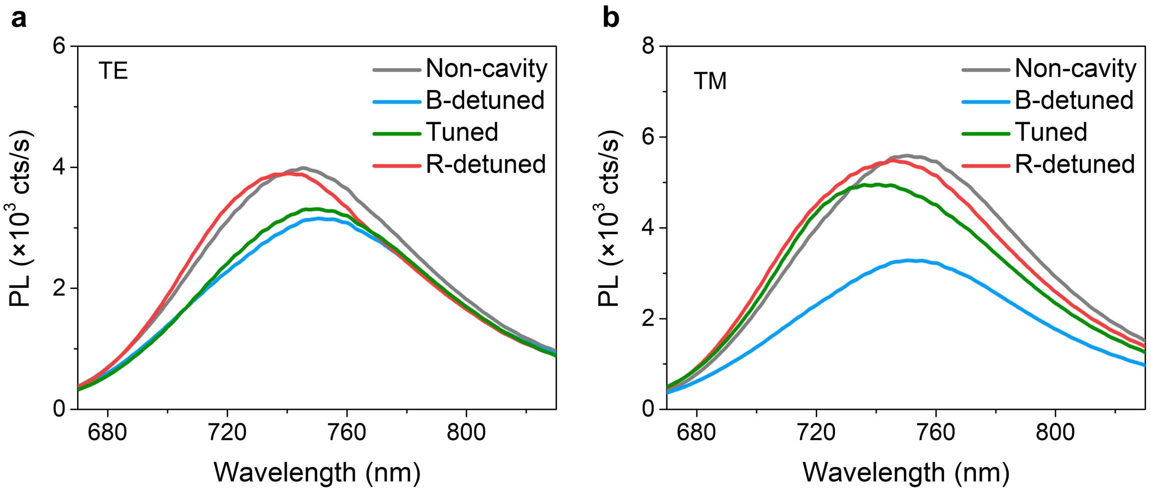


Figure S7: Averaged PL spectra in the range of -5 rad/$\mu$m $< k_{\|} <$ 5 rad/$\mu$m of the tuned and detuned systems. (a) TE-polarized PL, and (b) TM-polarized PL.

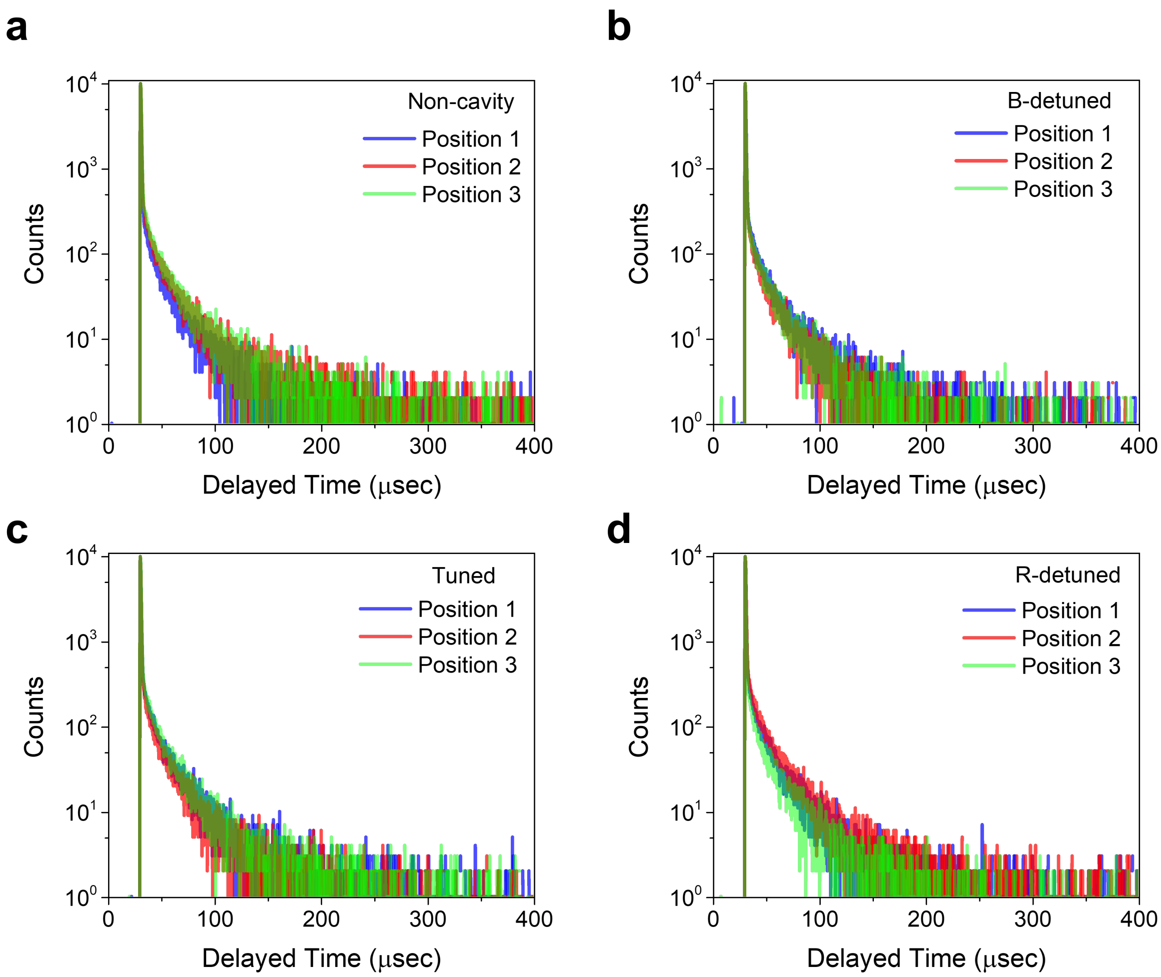


Figure S8: Excitation-position dependence of time-resolved time traces of the (a) non-cavity, (b) B-detuned, (c) tuned, and (d) R-detuned systems.

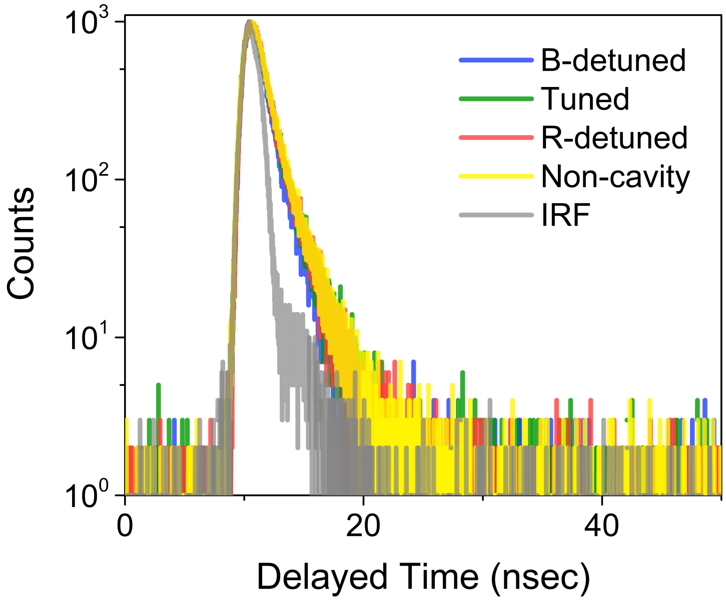


Figure S9: Nanosecond time-resolved PL. The excitation and detection wavelengths are 510 nm and 750 nm, respectively. IRF indicates the instrumental response function. The fitted averaged lifetime is 1.4 ns.

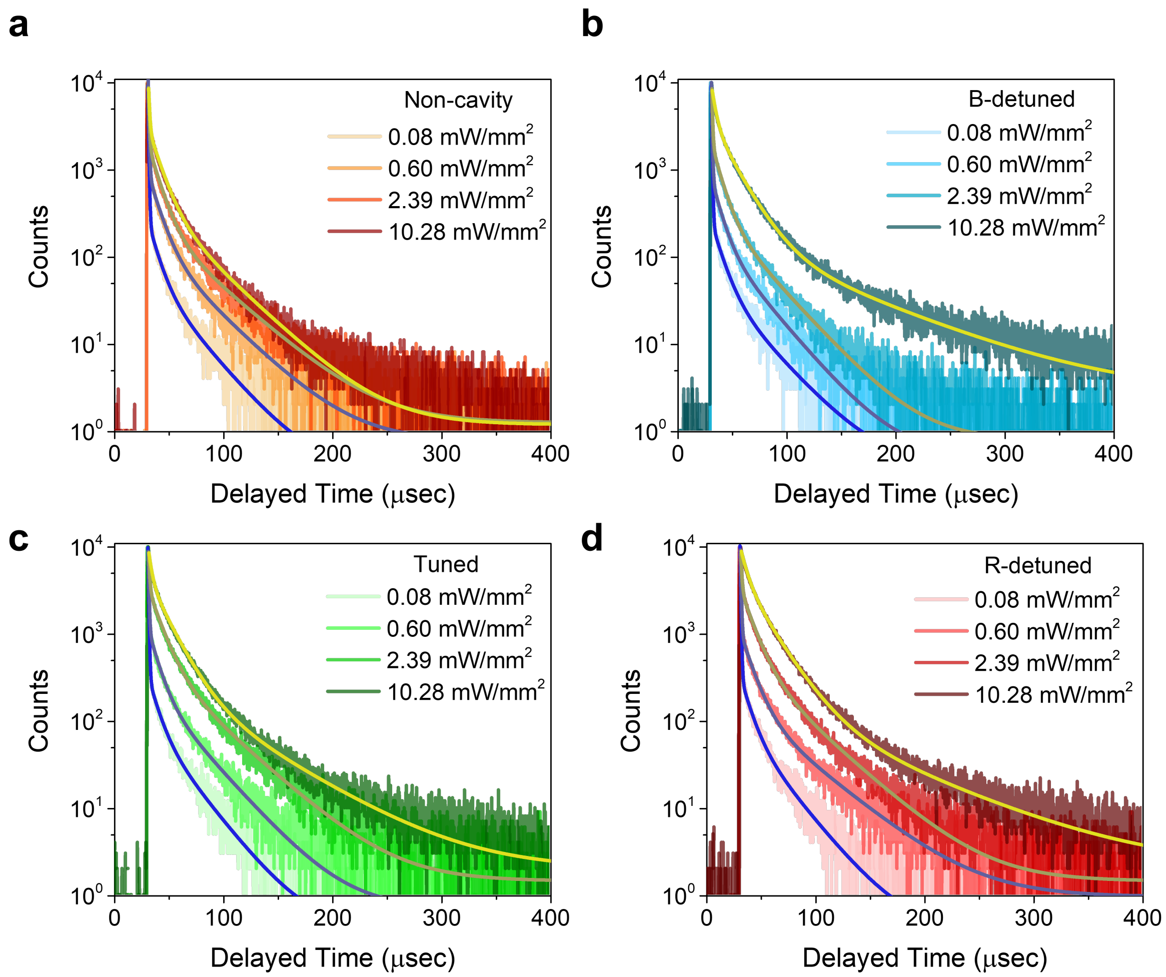


Figure S10: Power dependent microsecond time-resolved PL of (a) non-cavity, (b) B-detuned, (c) tuned and (d) R-detuned systems. The excitation and detection wavelengths are 535 nm and 750 nm, respectively. The solid lines are the fits to the measurements using a triple exponential decay function.

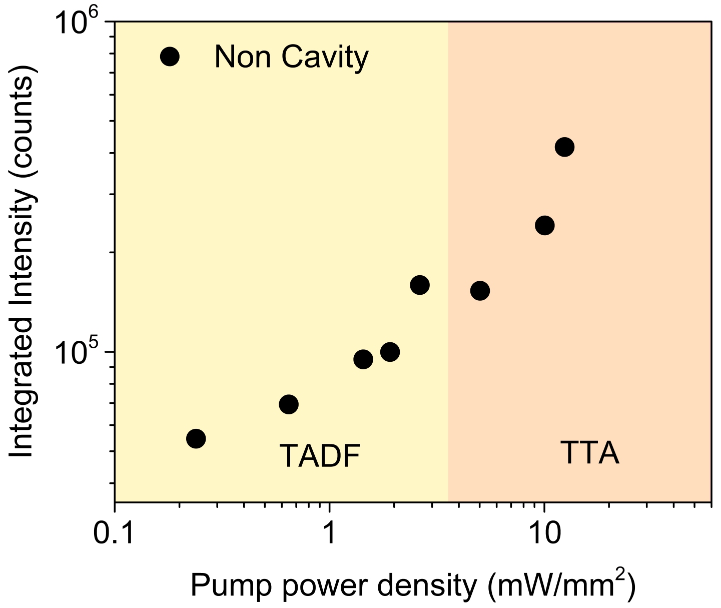


Figure S11: Power dependent steady-state PL intensity of the non-cavity system.

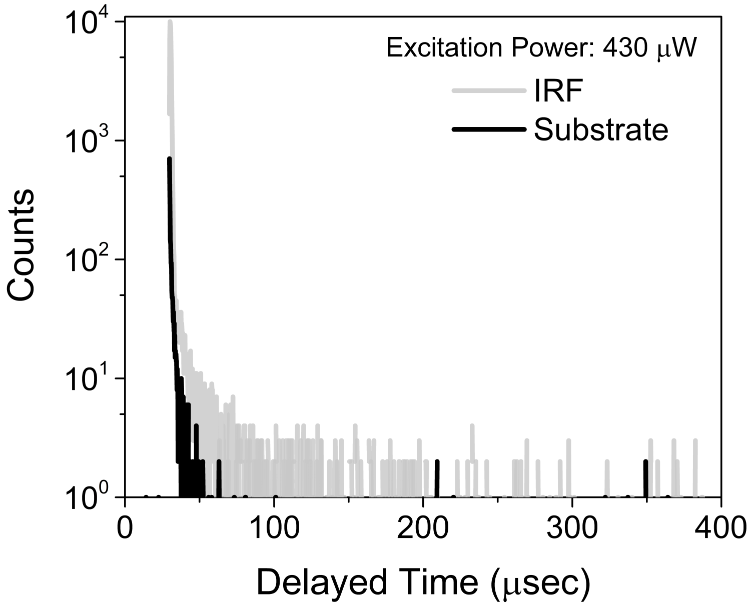


Figure S12: Microsecond time-resolved PL of the substrate. The excitation and detection wavelengths are 535 nm and 750 nm, respectively.

| Samples | Lifetime [μs] (pre-exponential factor) | | | |
|---|---|---|---|---|
| | 0.08 mW/mm$^2$ | 0.60 mW/mm$^2$ | 2.39 mW/mm$^2$ | 10.28 mW/mm$^2$ |
| Non-cavity | 0.557±0.005 (0.98);<br>8.0±0.4 (0.016);<br>29.3±1.0 (0.004) | 0.643±0.006 (0.94);<br>9.2±0.2 (0.054);<br>35.2±0.6 (0.006) | 0.707±0.010 (0.83);<br>9.2±0.1 (0.151);<br>39.2±0.5 (0.019) | 0.867±0.016 (0.70);<br>9.8±0.1 (0.253);<br>36.4±0.4 (0.047) |
| B-detuned | 0.555±0.005 (0.98);<br>8.8±0.4 (0.016);<br>31.4±1.2 (0.004) | 0.570±0.006 (0.94);<br>7.7±0.2 (0.047);<br>29.2±0.5 (0.013) | 0.648±0.010 (0.82);<br>8.2±0.1 (0.153);<br>31.7±0.4 (0.027) | 3.48±0.05 (0.59);<br>17.8±0.1 (0.391);<br>83.0±1.2(0.019) |
| Tuned | 0.538±0.005 (0.98);<br>7.8±0.4 (0.014);<br>27.7±1.1 (0.006) | 0.582±0.007 (0.90);<br>8.2±0.2 (0.076);<br>31.0±0.4 (0.024) | 0.886±0.018 (0.67);<br>10.7±0.1 (0.281);<br>38.1±0.4 (0.049) | 2.75±0.05 (0.53);<br>15.9±0.1 (0.433);<br>56.7±0.7 (0.037) |
| R-detuned | 0.578±0.005 (0.98);<br>8.2±0.4 (0.018);<br>27.9±0.8 (0.002) | 0.608±0.007 (0.91);<br>10.0±0.2 (0.073);<br>42.2±0.6 (0.017) | 0.894±0.018 (0.68);<br>10.8±0.1 (0.281);<br>38.3±0.4 (0.039) | 5.59±0.10 (0.52);<br>20.5±0.2 (0.461);<br>78.3±1.4 (0.019) |

Figure S13: Fitting parameters of the microsecond time-resolved PL. The unit of the lifetime is $\mu$s. The error bars result from the deviation between the measured value and the fits.
